# Impacts of gait freeze on quality of life in Parkinson’s disease, from the perspectives of patients and their carers

**DOI:** 10.1007/s11845-024-03673-x

**Published:** 2024-04-19

**Authors:** Padraig Cronin, Lucy M. Collins, Aideen M. Sullivan

**Affiliations:** 1 Department of Anatomy and Neuroscience, School of Medicine, University College Cork, Cork, Ireland; 2 Parkinson’s Disease Research Cluster, University College Cork, Cork, Ireland

**Keywords:** Caregivers, e-Health, Freezing of gait, Gait freeze, Motor symptoms, Non-motor symptoms, Parkinson’s disease, Quality of life

## Abstract

**Background:**

The World Health Organisation (WHO) reports that morbidity and mortality due to Parkinson’s disease (PD) are increasing faster than for other neurodegenerative conditions. People with Parkinson’s (PwP) present with a variety of motor symptoms, such as tremor, bradykinesia, and rigidity. Freezing of gait (FoG) is a significant motor symptom that manifests as temporary episodes of inability to move one’s feet, despite the intention to walk.

**Aims:**

This study examined the impact of FoG on quality of life (QoL) within an Irish cohort of PwP, from the perspectives of both PwP and their carers, using validated questionnaires that had been adapted for online use.

**Methods:**

PwP and their carers were recruited by outreach to the Irish Parkinson’s Community. Anonymous online questionnaires were distributed, which combined a demographic survey with several clinically validated surveys, including Freezing of Gait Questionnaire (FoG-Q), Parkinson’s Disease Questionnaire 8 (PDQ-8), and Parkinson’s Disease Carer Questionnaire (PDQ-C).

**Results:**

There was a strong correlation (*p* < 0.001) between severity of FoG and lower QoL among PwP. Significant correlation was also found between FoG severity and several motor symptoms, such as postural instability and difficulty with balance, and non-motor symptoms, such as cognitive changes and pain/discomfort. FoG severity correlated with disease progression. Significant correlation was also found between FoG and symptoms, as assessed from the perspective of the patients’ carers.

**Conclusions:**

This study shows that FoG is a significant detriment to the QoL of PwP, from the perspectives of patients and carers. This method of assessing FoG and QoL using online questionnaires has potential to enhance the reach and flexibility of this type of research. These findings will inform future studies on larger cohorts and highlight unmet clinical needs in PwP.

## Introduction

The World Health Organisation (WHO) has reported that disability and death due to Parkinson’s disease (PD) are increasing faster than for any other neurodegenerative condition [1]. PD is the second most prevalent neurodegenerative illness globally, affecting 1–2% of adults aged over 65 [2]. PD is a disease that affects mobility. It is caused by the degeneration of dopaminergic neurons of the substantia nigra pars compacta and the resulting decrease of dopamine neurotransmission in the caudate-putamen, regions of the brain that play critical roles in the control of motor function. As a result, patients present with motor symptoms such as tremor, rigidity, and slowing of gait. Freezing of gait (FoG) is one of the most debilitating and pervasive symptoms of PD; it can be defined as “brief, episodic absence or marked reduction of forward progression of the feet despite the intention to walk” [3]. People with Parkinson’s (PwP) often describe the phenomena as their feet being “glued” to the ground, blocking their ability to take a step [4]. It primarily affects gait, manifesting when starting to walk, in the middle of the movement, when turning or changing direction, when approaching obstacles, or in narrow spaces [5]. Once the FoG terminates, the patient can often walk smoothly and without impedance; however, over time, increasing incidences of FoG can result in postural instability and the confinement of a patient to a wheelchair [6, 7]. Clinical evaluation of FoG presents a challenge due to the variable frequency and presentation of the phenomena from moment to moment, affecting each patient differently. The frequency of FoG can be partially triggered by sensory and cognitive input. This presents a challenge for researchers in the measurement of the phenomena, due to a reliance on first-hand data from the patient and their carer in terms of their recall of the nature and frequency of the FoG episodes, as well as on clinical observations [5]. FoG often presents in the later course of PD, and analysis of cross-sectional studies indicates that FoG is present in 7% of patients within the first 2 years, 28% within 5 years, 39% within 10 years, and 58% after 10 years of the disease [4]. FoG has also been reported at very early stage PD, even before the commencement of treatment, and usually in short intervals of 1–2 s [8–10]. This increases drastically in the later stages of the disease, when FoG episodes last from several seconds to minutes [11]. As the frequency of FoG increases, it has been reported that the frequency of falls also increases [12]. While this was not observed in all prospective studies regarding falls in PwP, it is nonetheless an important risk factor that must be taken into account in the management of this patient cohort [13]. Furthermore, it is reported by patients that gait impairment and falls result in a loss of mobility, consequently reducing participation in social activities; this is an important factor that negatively impacts QoL [14, 15]. Embarrassment associated with entering a state of FoG in public can cause PwP to avoid social settings, thereby further reducing their QoL [16]. Additionally, falls due to FoG can cause hip fractures, particularly relevant due to the advanced age of a typical PwP, consequently leading to increased morbidity and mortality [7]. Analysis of data drawn from a previous study suggests that 25% of patients with PD will develop a hip fracture within 10 years of diagnosis [17], highlighting the danger of FoG within this cohort and the need for novel clinical management. Multiple methods of intervention have been suggested as means of improving and overcoming the frequency of FoG within PwP [18]. A promising area is the use of physical exercise, which has been shown to improve the mobility of PwP and consequently to reduce FoG [19–21]. A study which implemented a 2-week physical exercise regimen incorporating acoustic cueing reported a 22% reduction in the FoG score within a PwP cohort [22]. Another avenue of research that has demonstrated promising results is the use of external sensory cues, such as auditory or visual cues, to induce the termination of a FoG state and the reinstatement of normal walking [22–27]. A study conducted in Ireland showed positive effects of fixed rhythmic electrical cueing in a small cohort of PwP [28]. There are multiple types of cueing systems under investigation for use in PD, including auditory, visual, and somatosensory cues, each of which has advantages and limitations [29].

While the phenomenon of FoG has been identified by clinicians and researchers for several decades, it is not well understood, and optimisation of its clinical management is still being developed; this is an area of ongoing and increasing research. Our study examined gait freeze within an Irish cohort of PwP, with the aim of analysing the impacts of FoG on QoL as well as on motor and non-motor symptoms. It was an online study, which incorporated data from surveys completed by PwP, their personal caregivers, and their clinical caregivers. The use of such questionnaires, adapted for online remote use, provides greater flexibility for people to participate outwith clinical settings, and increases the reach of the study to larger and more diverse populations of patients and their carers. The perspective of carers has not been frequently included in previously-published studies of this type, and provides insight on the wider impacts of FoG on the families of PwP.

## Methods

**Fig. 1:**
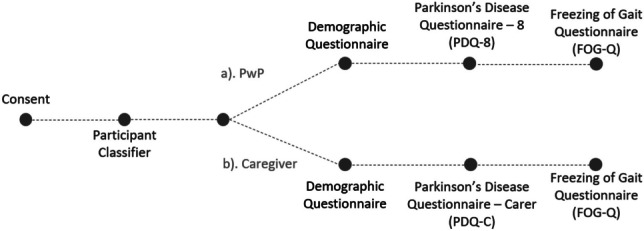
Survey design. PwP, person with PD; PDQ-8, The Parkinson’s Disease Questionnaire; PDQ-C, The Parkinson's Disease Questionnaire—Carer; FoG-Q, Freezing of Gait Questionnaire (modified for online use)

Ethical approval was granted through the University College Cork (UCC) Social Research Ethics Committee (SREC), log number 2023-104. The study was performed in accordance with the ethical standards as in the 1964 Declaration of Helsinki and its later amendments, or comparable ethical standards. In total, 121 participants (*n* = 88 PwP; *n* = 33 carers, of which 14 = family carers, 10 = clinical carers, 9 = prefer not to say) were recruited from the Parkinson’s community within the Republic of Ireland. The online study was designed and anonymised through the Qualtrics™ platform. Each participant was informed that they could withdraw from the survey at any time up until they had completed the survey. The participants were not pre-selected and represented a broad range of disease duration. Informed consent was obtained electronically from all participants. Data was gathered through remote online surveys to assess participants’ QOL and FoG, and included answers to questions on participant demographics as well as clinically-validated questionnaires: the Parkinson’s Disease Questionnaire (PDQ-8), the Parkinson's Disease Questionnaire—Carer (PDQ-C), and a version of the Freezing of Gait Questionnaire (FoG-Q) that was modified for remote online use. Survey questions were branched based on the participant, as outlined in Fig. 1.

Statistical analysis of the data was performed using IBM SPSS Statistics Version 28. Data was checked for normality. Prior to the commencement of data analysis, each response was examined and survey responses with < 50% completion were omitted, in order to minimise bias within the study. For examination of the QoL scores and FoG scores among PwP and carers, analysis was conducted by Pearson Bivariate Correlation. Correlation between motor/non-motor symptoms and FoG scores between groups was conducted by Mann-Whitney U testing, as homogeneity of variance was not met. Length of time experiencing PD vs age was examined using bivariate Spearman correlation, as the data satisfied the relevant assumptions for the model. Descriptive statistics were used to provide information about the sample and the QoL scores for the PwP and carer groups, with statistical significance defined at *p* < 0.05.

## Results

### Participants’ demographics

There was a total of 121 participants in this study, 97 of these submitted responses which had > 50% of the questions answered, and these responses were analysed. The majority of PwP were retired (65.8%), male (50.7%), aged 70–74 years (28.8%), and of White Irish ethnicity (87.7%). The majority of carers were employed full time (39.1%), female (91.3%), aged 65–69 years (29.2%), and of White Irish ethnicity (90.9%) (Table 1). These carers included both direct caregivers with a close personal relationship to the PwP (partner, child, etc.; *n* = 14) and clinical caregivers with a professional relationship to the PwP (nurses, doctors, physiotherapists, etc.; *n* = 10).

**Table 1:** Participants’ demographics

**Demographics**	**PwP**	**Caregivers**
Age	70-74 years: 28.8% (*n* = 73)	65-69 years: 29.2% (*n* = 24)
Gender (male)	50.7% (*n* = 73)	8.7% (*n* = 23)
**Race (White Irish)**	87.7% (*n* = 73)	90.9% (*n* = 22)
Employment status (retired)	65.8% (*n* = 73)	34.8% (*n* = 23)
Age of diagnosis (60–64 years old)	28.8% (*n* = 73)	-
Length of time experiencing PD symptoms (7–9 years)	22.5% (*n* = 71)	26.1% (*n* = 23)
Participation in clinical trials (yes)	20.0% (*n* = 70)	14.3% (*n* = 21)*
Participation in procedures (yes)	13.9% (*n* = 72)	9.1% (*n* = 22)*
Family history of PD (yes)	29.6% (*n* = 71)	19.0% (*n* = 21)*
**Experience of gait freeze within the past month**	27.9% (*n* = 68)	75% (*n* = 20)*

The highest percentage response is shown for each question

*M* male, *PwP* people with Parkinson’s, *PD* Parkinson’s disease

*Responses regarding the PwP about whom the caregiver is responding

### Impact of FoG on QoL of PwP and carers

The FOG-Q score was compared against both the PDQ-8 and PDQ-C single index scores, to investigate any significant correlation between the severity of the FoG state and the QoL of both the PwP and carer. This included comparison of the cumulative score of the FOG-Q as well as of each individual question of the FOG-Q. A strong correlation was found between the severity of FoG and the QoL experienced by PwP, as measured by the PDQ8 scores (*p* < 0.001; Table 2). This was the case for the total FOG-Q score as well as for each question of the FOG-Q (*p* < 0.001 level of significance for each; Table 2). This was not the case for the carers group, where there was no significant correlation between the QoL as measured by the PDQ-C score and the severity of FoG experienced by their PwP (*p* = 0.290370; Table 2).

**Table 2:** Impact of FoG on QoL of PwP and carers

	**Pearson correlation**	**Mean value**	**Standard error**	**Significance** **(2-tailed)**
**PDQ8 single index**				
*vs total FoG-Q score (n* = *45)*	(+) 0.746	5.6167	0.51426	< 0.001
*vs FoG-Q Q1 score (n* = *48)*	(−) 0.665	0.7121	0.05616	< 0.001
*vs FoG-Q Q2 score (n* = *46)*	(+) 0.682	1.6349	0.15397	< 0.001
*vs FoG-Q Q3 score (n* = *48)*	(+) 0.652	1.1385	0.13671	< 0.001
*vs FoG-Q Q4 score (n* = *48)*	(+) 0.582	1.1061	0.16603	< 0.001
*vs FoG-Q Q5 score (n* = *47)*	(+) 0.567	1.0000	0.16815	< 0.001
**PDQC single index**				
*vs total FoG-Q score (n* = *12)*	(−) 0.332892	8.4000	0.74833	0.290370
*vs FoG-Q Q1 score (n* = *12)*	(+) 0.203398	1.25	0.099	0.526052
*vs FoG-Q Q2 score (n* = *12)*	(−) 0.292816	3.67	0.252	0.331601
*vs FoG-Q Q3 score (n* = *13)*	(+) 0.111915	1.57	0.130	0.715856
*vs FoG-Q Q4 score (n* = *13)*	(−) 0.200798	3.67	0.279	0.510673
*vs FoG-Q Q5 score (n* = *13)*	(−) 0.191299	3.05	0.320	0.531272

Within the PwP cohort, there was a strong correlation (*P* < 0.001) between each section of the FoG-Q and QoL as measured by PDQ8. Within the carer cohort, there was minimal correlation between the QoL of the carer and the FoG score of their PwP. Analysis was conducted using Pearson bivariate correlation, with statistical significance defined at *p* < 0.05

*PwP* people with Parkinson’s disease, *PD* Parkinson’s disease, *PDQ-8* Parkinson’s Disease Questionnaire, *PDQ-C* Parkinson’s Disease Questionnaire—Carer, *FoG-Q* modified Freezing of Gait Questionnaire

### Correlations between severity of FoG and motor and non-motor symptoms

**Fig. 2:**
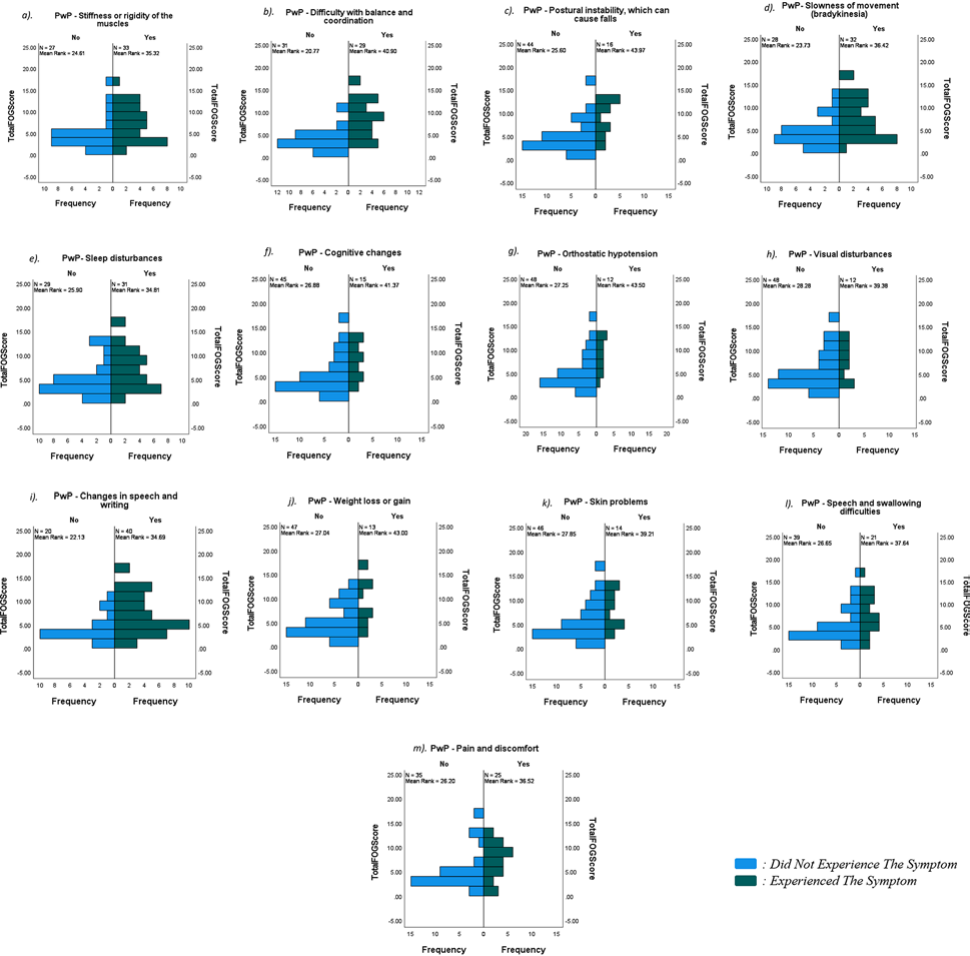
Correlations between severity of FoG and motor and non-motor symptoms in PwP, as indicated by PwP. Graphs demonstrating correlations between FoG and motor and non-motor symptoms. Data shown are from questionnaires completed by PwP, and only those symptoms with statistically significant correlations are displayed. Analysis of correlations between motor/non-motor symptoms and FoG scores between groups was conducted by Mann-Whitney U testing; statistical significance was defined at *p* < 0.05. PwP, people with Parkinson’s disease.; PD, Parkinson’s disease; FoG-Q, modified Freezing of Gait Questionnaire

**Fig. 3:**
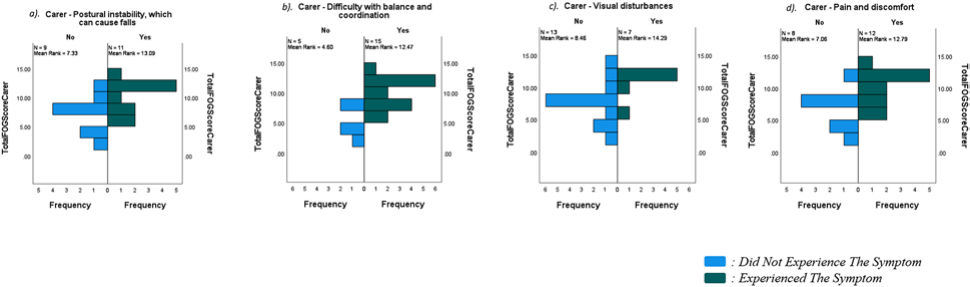
Correlations between severity of FoG and motor and non-motor symptoms in PwP, as indicated by carers. Graphs demonstrating correlations between FoG and motor and non-motor symptoms. Data shown are from questionnaires completed by carers, and only those symptoms with statistically significant correlations are displayed. Analysis of correlations between motor/non-motor symptoms and FoG scores between groups was conducted by Mann-Whitney U testing; statistical significance was defined at *p* < 0.05. PwP, People with Parkinson’s disease; PD, Parkinson’s disease; FoG-Q, modified Freezing of Gait Questionnaire

The FoG-Q was compared against both motor and non-motor symptoms for each cohort, to determine whether there were any significant correlations between the severity of the FoG state and the prevalence of the specific symptoms. It must be noted that data was collected for each of 13 separate motor symptoms and 23 separate non-motor symptoms; however, only those with statistical significance are reported in Table 3. The strongest correlation with FoG was found with difficulty with balance and coordination (*p* < 0.001; Table 3, Fig. 2B) and with postural instability (*p* < 0.001; Table 3, Fig. 2C). There were also significant correlations between FoG and stiffness/rigidity (*p* = 0.017), bradykinesia (*p* = 0.005), changes in speech/writing (*p* = 0.008), sleep disturbance (*p* = 0.047), cognitive changes (*p* = 0.005), visual disturbance (*p* = 0.048), orthostatic hypotension (*p* = 0.004), pain/discomfort (*p* = 0.023), speech/swallowing difficulties (*p* = 0.019), weight loss/gain (*p* = 0.003), and skin problems (*p* = 0.032) (*p* < 0.05; Table 3, Figs. 2 and 3).

**Table 3:** Correlations between severity of FoG and motor and non-motor symptoms

**Total FoG score**	**Significance (2-tailed)**
**PwP data**	
***Motor symptoms***	
*vs stiffness or rigidity*	0.017
*vs difficulty with balance and coordination*	< 0.001
*vs postural instability*	< 0.001
*vs slowness of movement (bradykinesia)*	0.005
*vs changes in speech or writing*	0.008
***Non-motor symptoms***	
*vs cognitive changes*	0.005
*vs orthostatic hypotension*	0.004
*vs visual disturbance*	0.048
*vs pain and discomfort*	0.023
*vs speech and swallowing difficulties*	0.019
*vs weight loss or gain*	0.003
*vs skin problems*	0.032
*vs sleep disturbance*	0.047
**Carer data**	
***Motor symptoms***	
*vs difficulty with balance and coordination*	0.008
*vs postural instability*	0.031
*Non-motor symptoms*	
*vs visual disturbance*	0.037
*vs pain and discomfort*	0.031

Table demonstrating the significant correlations between FoG and motor and non-motor symptoms. This was conducted for both PwP and carers, and data from both groups was combined. Only those symptoms that correlated highly significantly with FoG are displayed in this table. Analysis of correlations between motor/non-motor symptoms and FoG scores between groups was conducted by Mann-Whitney U testing; statistical significance was defined at *p* < 0.05

*PwP*, people with Parkinson’s disease; *PD*, Parkinson’s disease; *FoG-Q*, modified Freezing of Gait Questionnaire

### Correlation between disease duration and severity of FoG

To investigate whether there was an impact of disease duration and the severity of FoG, the total FoG score was assessed in PwP at different disease stages. A strong correlation between length of disease and severity of FoG was found in the data from the questionnaires completed by the PwP cohort (*p* < 0.001; Table 4), but not in those completed by the carer cohort (*p* = 0.304; Table 4).

**Table 4:** Correlation between disease duration and severity of FoG

**Total FOG score**	**Pearson correlation**	**Significance (2-tailed)**
***vs length of time experiencing PD—PwP cohort***	(+) 0.457	< 0.001
***vs length of time experiencing PD—carer cohort***	(+) 0.242	0.304

Correlations between total FoG score and length of time experiencing PD, data from questionnaires completed by the PwP and carer cohorts. Analysis of correlations between length of time experiencing PD and FoG severity was conducted by Spearman rho testing; statistical significance was defined at *p* < 0.05

*PwP* people with Parkinson’s disease, *PD* Parkinson’s disease, *FoG-Q* modified Freezing of Gait Questionnaire

## Discussion

The incidence of FoG as reported by PwP in this remote online assessment was reflective of a previously-published study, which reported that 50.6% of PwP experience FoG [30]. In our study, the incidence of FoG in PwP from a caregiver’s perspective was significantly higher than that reported by the patients themselves (75% vs 27.9%). This may be reflective of the wider exposure of healthcare workers to PwP during the later stages of the disease progression, at which stage FoG would be more frequent than at earlier disease stages, so there would be a higher likelihood of clinical caregivers encountering FoG in their patients. The average age of diagnosis as reported by PwP was within the range of 60–64 years old. Our comparison of length of time experiencing PD and severity of FoG as reported by the PwP cohort demonstrated a positive correlation; this indicates the gradual worsening of FoG with the progression of the disease course. However, data from the carer cohort did not show significant correlation between length of disease and FoG severity. This may reflect the small sample size of this cohort, particularly the low number (*n* = 14) of direct caregivers, and so further studies with larger numbers of carers would be beneficial to explore this discrepancy.

Our study found a slightly higher history of familial PD in this cohort of patients (i.e. 29.6% and 19%, respectively, as reported by PwP and carers) than the global incidence (5–15%) reported in current literature [31, 32]. This may be reflective of a higher prevalence of familial PD within an Irish cohort, although larger studies would be needed to examine this. Despite the higher prevalence of familial PD within this study, no significant correlation between familial PD and FoG severity could be drawn from the data gathered. 

We found a strong correlation across each question of the FoG questionnaire with QoL of PwP, as assessed by the PDQ8 single index. Each individual question demonstrated a positive correlation, except for Q1, which pertained to the prevalence of FoG and showed a negative correlation. This strongly corroborates a previous study showing an association between severity of FoG and decreased QoL in PwP [16], but this is the first study to demonstrate this within an Irish cohort. In comparison to the strong correlation between FoG and QoL in the responses of the PwP cohort, there was no significant association found from the perspective of the cohort of carers. This may be a result of the small study population.

In addition to measures of QoL, our study analysed both motor and non-motor symptoms that are characteristic of PD, to investigate whether there were any associations between FoG severity and specific symptoms. Detailed examination of the PwP responses regarding individual symptoms revealed strong positive correlations between FoG severity and 6 of the 15 motor symptoms, and 7 out of the 23 non-motor symptoms. The main motor symptoms affected included difficulty with balance and coordination, postural instability, and bradykinesia. These findings are all consistent with prior reports [33] and would account for the higher rate of falls reported in PwP who experience FoG [34]. This was supported by data from the carer cohort, which identified a significant relationship between FoG severity and postural instability in their patients, and difficulty with balance and coordination. A unique finding in this study was the positive correlation between severity of FoG and stiffness or rigidity. Previous studies have not reported evidence of a link between these two phenomena [9, 35]; however, this difference in findings may be due to the older demographic within our study compared to previous studies. Older PwP tend to present with a higher prevalence of rigidity, and the correlation found here between FoG severity and disease progression would align with the association between FoG severity and stiffness or rigidity. Another motor symptom which showed a significant correlation with FoG severity was changes in speech or writing; these findings are consistent with the literature, indicating a common pathophysiological connection between FoG and speech. This association has been hypothesised to occur as a result of dysfunction in PD of the cognitive control network, which involves both the frontal and parietal areas, leading to both speech and gait difficulties [36].

As well as motor symptoms, several non-motor symptoms correlated very highly with FoG severity in our study. For example, visual disturbance positively correlated with severity of FoG, from the perspectives of both PwP and their carers. This may be due to worsening of these visual symptoms, in parallel with worsening of FoG, as the disease progresses [37]. There was also a significant association reported between severity of FoG and sleep issues. Several theories exist regarding this association, including the theory that disruption of circadian rhythms in PD patients can lead to difficulties in gait initiation [38], and that PD-induced degeneration of the locus coeruleus and the ascending reticular activating system can lead to increased daytime sleep and related sleep disturbance [39], which may impact upon gait. Larger studies support these findings and indicate a direct link between sleep disturbance and FoG [40], demonstrating that our remotely-assessed cohort is reflective of previous in-person cohorts studied. We also found a strong correlation between FoG severity and pain and discomfort, and this was reported by both the PwP themselves as well as their carers, and is in line with previous studies [41–43]. In PD, neurodegeneration is not confined to the dopaminergic system but can also occur in other neuronal systems, including serotonergic, cholinergic, and peptidergic, accounting for the wide variety of motor and non-motor symptoms [42]. Additionally, connections between the frontal cortex, cerebellum, and thalamus are also decreased due to FoG in PD [44]. These factors, coupled with the pain caused by the movement associated with FoG, could explain the correlation between FoG severity and pain and discomfort, as well as the association of FoG with sleep problems.

Another non-motor symptom that was found to be significantly correlated with FoG was orthostatic hypotension, which is a well-documented symptom of PD and can result from several aetiologies. The most common reason is neurogenic, whereby it occurs as a result of the progressive autonomic failure seen in PD [45, 46]. Non-neurogenic causes can include the side-effects of certain medications such as levodopa [47] and dopaminergic agonists (e.g. pramipexole) [48, 49] which are used as standard PD treatment, as well as other drugs used to treat depression, hypertension, or bladder dysfunction [50], all of which commonly occur in PwP and tend to worsen with disease progression. Thus, our result of a correlation between orthostatic hypotension and severity of FoG is consistent with our finding of increasing severity of FoG with disease progression. Another non-motor symptom that demonstrated a strong correlation within this study was weight loss or gain. Weight change in PwP is a complex phenomenon which occurs due to a variety of reasons. Medication plays a notable role in weight gain in PwP. Dopamine agonists are widely used in the treatment of severe FoG in PwP [51], but these drugs are associated with compulsive eating behaviour due to the activation of mesolimbic dopaminergic pathways, which may lead to weight gain in some cases [52]. Conversely, weight loss may also be attributed to malnutrition and correlates heavily with the severity of the disease [53], which, as discussed above, has been shown to correlate highly with FoG in our study. Skin problems were also flagged as a symptom that strongly correlated with FoG severity. Skin conditions, particularly seborrheic dermatitis, rosacea, bullous pemphigoid, and melanoma, each have an increased incidence in PwP [54] and have been attributed to dysregulation of the autonomic nervous system [55], or to inhibition of tyrosine hydroxylase by α-synuclein [56]. Both phenomena occur progressively throughout the course of the disease and may account for the correlation with severity in FoG found in our study. Each of the associations found in the data from our study was consistent with our first-hand observations during visits to meetings of various support groups of PwP, and from discussions with healthcare workers throughout the period of the study.

It must be acknowledged that there were certain limitations to this study. Firstly, the FoG was adapted and shortened to improve the recruitment and retention of participants to this study. The standard FoG questionnaire is normally administered in a clinical setting, scoring an individual with PD both in ‘on’- and ‘off’-medication periods. Secondly, our survey was administered to both PwP and carers and asked them to recall the prevalence or frequency of variables being examined. This may have introduced recall bias which could affect the results of the study. Thirdly, this was a cross-sectional study with small cohorts for both PwP and carers, which may lead to underrepresentation.

Our study not only answered the original questions posed, but also provided several avenues for future research to better understand gait freeze in PwP. For example, since this data was analysed from a small cohort (*n* = 97), future research would benefit from gathering data from a larger cohort to better understand the phenomena. One key area of feedback drawn from this study was that healthcare workers were unable to fully explore the depth and frequency of symptoms seen within PwP, due to the fact that they routinely work with a large number of patients. Future research would benefit from a more comprehensive study to examine not only FoG but also other PD symptoms. This could identify specific trends to help healthcare to identify signs indicative of FoG at earlier disease stages, allowing early intervention. Our study made use of an online survey system with the aim of increasing accessibility, reducing paper usage, and improving the efficiency of data collection within a short time frame. While this method has proven to be very useful for this study, some older PwP found it difficult to use the technology needed to complete the study. Future research could include a paper-based option for participants, as an alternative to the online surveys. While this method would take longer to complete, it would capture a larger cohort of PwP participants and improve representation. Throughout the promotion of the study among PwP at various meetings, it was evident that PD is very much a unique condition that presents differently for each PwP. Future studies could incorporate interviews to identify nuanced details and themes which may not be collected in a survey-based study.

In conclusion, our study found that FoG has a significant negative impact on the QoL of PwP. FoG severity tends to increase with the length of time from PD diagnosis. Ours is the first study to examine the relationship between FoG and QOL within an Irish cohort of PwP. In addition to collecting data from PwP themselves, our study also included an assessment of the impact of FoG on QoL and on motor and non-motor symptoms from the perspective of the patients’ primary caregivers, providing an objective perspective from a person with daily contact with, and insight to, the patients’ experiences. Severity of FoG was significantly associated with a combination of motor and non-motor symptoms, including stiffness or rigidity, postural instability, difficulty with balance and coordination, bradykinesia, cognitive changes, sleep and speech disturbances, and generalised pain and discomfort. This study validates the use of online remote questionnaires to examine the impact of FoG on QoL and symptoms in PwP. The use of such an online assessment method has great potential to increase participation in research on the parts of both patients and their carers, as they can save time and expense by not travelling to clinical sites. Increased engagement by those living in rural areas, or by those in full-time employment, is another positive outcome of this type of online research study.

## Appendices

### Appendix 1 All PwP data—FoG vs motor and non-motor symptoms

**Table:**

**Total FoG score**	**Significance (2-tailed)**
***Motor symptoms***	
*vs stiffness or rigidity*	**0.017**
*vs tremors or shaking*	0.188
*vs difficulty with balance and coordination*	** < 0.001**
*vs postural instability*	** < 0.001**
*vs slowness of movement (bradykinesia)*	**0.005**
*vs changes in speech or writing*	**0.008**
*vs dystonia*	0.467
*vs shuffling walk*	0.798
*vs dyskinesia*	0.933
*vs walking too fast after freeze*	-
***Non-motor symptoms***	
*vs depression and anxiety*	0.948
***vs sleep disturbance, including excessive daytime sleepiness, insomnia, or restless leg syndrome***	**0.047**
*vs loss of sense of smell*	0.177
*vs constipation*	0.056
*vs cognitive changes*	**0.005**
*vs excessive sweating*	0.249
*vs fatigue or lack of energy*	0.140
*vs orthostatic hypotension*	**0.004**
*vs visual disturbance, including blurry vision or double vision*	**0.048**
*vs sexual dysfunction*	0.125
*vs pain and discomfort*	**0.023**
*vs speech and swallowing difficulties*	**0.019**
*vs mood swings and emotional changes*	0.292
*vs weight loss or gain*	**0.003**
*vs skin problems, including dry skin and rashes*	**0.032**
*vs nocturnal dysuria*	0.933
*vs generalised low blood pressure*	0.667
*vs urinary incontinence*	0.933
*vs heat intolerance*	-
*vs apathy*	-
*vs irritability*	0.367
*vs vertigo*	0.733
*vs emotional changes*	0.667
***vs reduced sense of smell***	0.614
***vs difficulty with swallowing***	0.250
***vs non-motor symptoms, including depression, anxiety, apathy, and cognitive impairment***	0.330

### Appendix 2 All carer data—FoG vs motor and non-motor symptoms

**Table:**

**Total FoG score**	**Significance (2-tailed)**
***Motor symptoms***	
*vs stiffness or rigidity*	0.494
*vs tremors or shaking*	0.552
*vs difficulty with balance and coordination*	**0.008**
*vs postural instability*	**0.031**
*vs slowness of movement (bradykinesia)*	0.064
*vs changes in speech or writing*	0.384
*vs dystonia*	0.700
*vs shuffling walk*	-
*vs dyskinesia*	-
*vs walking too fast after freeze*	1.000
***Non-motor symptoms***	
*vs depression and anxiety*	0.571
*vs sleep disturbance*	0.370
*vs loss of sense of smell*	0.393
*vs constipation*	0.122
*vs cognitive changes*	0.734
*vs excessive sweating*	0.485
*vs fatigue or lack of energy*	0.201
*vs orthostatic hypotension*	0.588
*vs visual disturbance, including blurry vision or double vision*	**0.037**
*vs sexual dysfunction*	0.119
*vs pain and discomfort*	**0.031**
*vs speech and swallowing difficulties*	0.280
*vs mood swings and emotional changes*	0.824
*vs weight loss or gain*	0.612
*vs skin problems*	0.699
*vs nocturnal dysuria*	-
*vs generalised low blood pressure*	-
*vs urinary incontinence*	-
*vs heat intolerance*	-
*vs apathy*	-
*vs irritability*	-
*vs vertigo*	-
*vs emotional changes*	-
*vs difficulty with swallowing*	0.056
*vs non-motor symptoms, including depression, anxiety, apathy, and cognitive impairment*	0.473
*vs reduced sense of smell*	0.757
*vs difficulty with swallowing*	0.056

## Abbreviations

FoG: Freezing of gait
FoG-Q: Freezing of Gait Questionnaire
PD: Parkinson’s disease
PDQ-C: Parkinson’s Disease Carer Questionnaire
PDQ-8: Parkinson’s Disease Questionnaire 8
PwP: People with Parkinson’s
QoL: Quality of life
